# INSR novel mutations identified in a Chinese family with severe INSR-related insulin resistance syndromes: A case report

**DOI:** 10.1097/MD.0000000000032266

**Published:** 2022-12-09

**Authors:** Lu Yu, Fang Yu, Zongrui Ma, Huilin Lu, Jian Luo, Ting Sun, Qin Liu, Shenglian Gan

**Affiliations:** a Department of Endocrinology and Metabolism, The First People’s Hospital of Changde City, Changde, Hunan, P. R. China; b Department of Ophthalmology, The First People’s Hospital of Changde City, Changde, Hunan, P. R. China.

**Keywords:** Donohue syndrome (DS), insulin receptor gene, insulin resistance, Rabson–Mendenhall syndrome (RMS)

## Abstract

Severe insulin receptor gene (*INSR*)-related insulin resistance syndromes (SIR) include Donohue syndrome (DS), Rabson–Mendenhall syndrome (RMS), and type A insulin resistance. The incidence of DS is about 1 in 4 million births. We identified novel *INSR* mutations (c.2246delG and c.2646 + 5G > A) in a patient with SIR, which expanded the variant spectrum and helped to improve the understanding of the diagnosis and treatment of this condition.

**Patient concerns:**

A 10-year-old Chinese boy was admitted to the hospital for deepening skin color. He presented with growth retardation, peculiar facial features, acanthosis nigricans, hypertrichosis, extremely high insulin levels, fasting hypoglycemia, and postprandial hyperglycemia, Whole-exome gene testing suggested compound heterozygous mutations in INSR (c.2246delG and c.2646 + 5G > A).

**Diagnosis:**

The diagnosis was SIR. What’s more, based on the phenotypic and biographical results, this child did not present typical RMS and DS but rather an intermediate phenotype between the 2 conditions.

**Interventions:**

On the basis of a sensible diet and exercise, the patient was prescribed metformin (250 mg) at breakfast and lunch, which was increased to 500 mg after 1 month.

**Outcomes:**

After 2 months of treatment, the patient’s glycated hemoglobin (HbA1c) levels decreased to 6% but his insulin resistance did not improve significantly.

**Lessons:**

In children who are not obese but with severe insulin resistance, growth retardation, hirsutism, and hyperglycemia, genetic testing should be performed for early diagnosis, active treatment, and follow-up.

## 1. Introduction

Abnormalities in the insulin receptor gene (*INSR*) can cause genetic insulin resistance, including DS, Rabson–Mendenhall syndrome (RMS), and type A insulin resistance, which are identified by the severity of the receptor function defect and unique physical characteristics of the affected individual.^[1]^ DS, with an incidence of approximately 1 in 4 million births, is the most severe syndrome.^[2]^ It is characterized by intrauterine and postnatal growth retardation, coarse facial features, severe insulin resistance, and fasting hypoglycemia. Most patients with DS die within the first 2 years of life. Patients with RMS have a relatively mild phenotype and it is often difficult to distinguish between the 2 syndromes given their overlapping symptoms. Thus, cases that lead to neonatal or early childhood death are usually classified as DS, while those that do not lead to such early death are usually diagnosed as RMS.^[2,3]^ We report the case of a male child with 2 novel *INSR* mutations (c.2246delG and c.2646 + 5G > A) identified by whole-exome genetic testing who was diagnosed with severe *INSR*-related insulin resistance syndromes (SIR).

## 2. Case presentation

**Figure 1.**
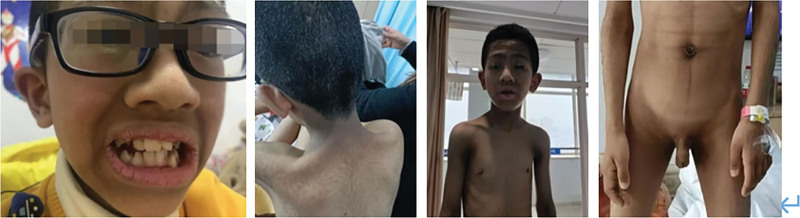
Clinical characteristics of the patient: dense curly hair; coarse facial features; thickened lips; irregular teeth; acanthosis nigricans in the neck, axillae, elbow sockets, and groin; and decreased subcutaneous fat.

**Figure 2.**
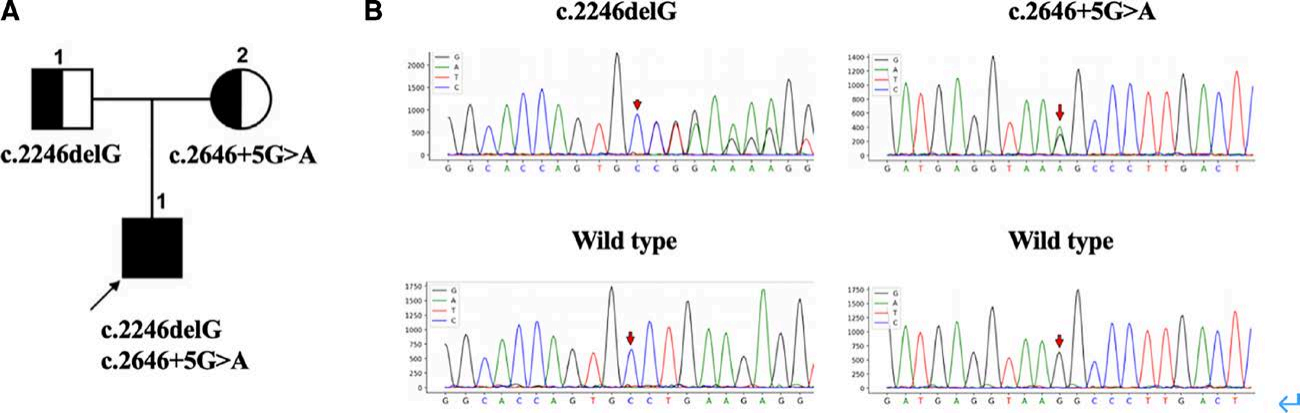
(A) Pedigree diagram of the patient with SIR and his family. (B) INSR variations identified by Sanger sequencing. INSR = insulin receptor gene.

The patient was a 10-year-old Chinese boy whose parents were young, healthy, and not consanguineously married. The patient was their first child and was delivered by cesarean section at full term with a birthweight of 3.2 kg. He had no history of intrauterine growth retardation. He was born with significant body hair and dark skin. At 1 year of age, he showed significant hyperpigmentation in the skin folds of his neck, elbows, and the base of his thighs, with no pubic or axillary hair growth. At 2 years of age, his parents noticed hearing loss and he learned to speak after cochlear implant surgery. At 3 years of age, he developed vision loss and was examined for high myopia. He was sent to the Department of Pediatric Endocrinology at 9 years of age for deepening skin color. After obtaining the consent of the patient and his family members, genetic analysis of *INSR* was performed and the patient was diagnosed with DS with a normal fasting blood sugar level. The patient’s family visited the local hospital 1 month ago for a skin mass on the patient’s buttocks. At that time, his glycated hemoglobin (HbA1c) level was 6.25% and his intravenous random blood glucose concentration was 13.12 mmol/L; therefore, the patient was referred to our hospital. The patient weighed 32 kg (SD −2.0) and was 130 cm in height. Physical examination revealed a variety of phenotypic abnormalities, including dense curly hair; large low-lying ears; coarse facial features; thickened lips; irregular teeth; acanthosis nigricans in the neck, axillae, elbow sockets, and groin; and decreased levels of subcutaneous fat (Fig. 1). Laboratory tests showed elevated HbA1c levels (6.8%). The oral glucose tolerance test and insulin and C-peptide release tests indicated severe hyperinsulinemia (Table 1). Assessments for anti-insulin antibody, protein tyrosine phosphatase autoantibody (IA2-Ab), and anti-glutamate decarboxylase antibody were all negative. The patient’s insulin-like growth factor-1 (IGF-1) levels were decreased (23.8 ng/ml, −2.9 SD) and radiology of his left hand showed a bone age of 10 years. Hormonal data showed that he was in the prepubertal stage. No significant abnormalities in adrenal hormones, thyroid hormones, 17 hydroxyprogesterone, and electrolytes were observed. His triglyceride and cholesterol levels were normal. Abdominal ultrasound showed slight enlargement of both kidneys. Continuous glucose monitoring showed fluctuations in fasting glucose levels from 4.1 to 5.6 mmol/l and 2 hours postprandial glucose levels from 4.7 to 17.9 mmol/l. Genetic analysis of *INSR* revealed a novel heterozygous mutation in exon 11, c.2246delG (p.G749fs); his father showed a heterozygous mutant genotype, while his mother showed a wild type locus (Fig. 2). According to the American College of Medical Genetics and Genomics (ACMG) guidelines for gene mutation interpretation, this locus was consistent with 3 lines of evidence and graded as pathogenic. A new heterozygous mutation was identified in exon 12, c.2646 + 5G>A, for which the patient’s father was wild type and his mother showed a heterozygous mutant locus. Based on the ACMG guidelines, this locus was consistent with 4 lines of evidence and was graded as likely pathogenic. These 2 mutations were not included in the Human Gene Mutation Database professional and Clinvar databases. The population frequency of the locus was not included. The mutations were also not included in the gnomAD database for East Asian populations. The 2 mutations were verified by family lineage to be compound heterozygous mutations, consistent with a recessive inheritance pattern. Therefore, the patient was diagnosed with SIR. Based on the phenotypic and biographical results, this child did not show the typical presentations of RMS and DS, but rather an intermediate phenotype between the 2 conditions. The patient was prescribed metformin (250 mg) at breakfast and lunch, which was increased to 500 mg after 1 month. After 2 months of treatment, the patient’s HbA1c levels decreased to 6% but his insulin resistance did not improve significantly.

**Table 1:** OGTT results.

Time (h)	Blood Glucose (mmol/l)	Insulin (pmol/L)	C-Peptide (nmol/L)
0	5.05	>2089.5	1.46
0.5	13.03	>2089.5	3.46
1	4.96	>2089.5	3.64
2	3.92	>2089.5	1.39
3	3.89	1780.34	1.13

OGTT: oral glucose tolerance test.

## 3. Discussion

Hereditary insulin resistance syndromes include genetic syndromes caused by *INSR* mutations, SHORT syndromes caused by *PIK3R1* abnormalities, and conditions caused by *AKT2*, *TBC1D4*, or *PRKCE* abnormalities.^[1]^

INSR is a cell surface heterotetrameric glycoprotein and is a member of the class II receptor tyrosine kinase superfamily. It comprises 2 extracellular α subunits and 2 transmembrane β subunits joined by disulfide bonds, in which the α subunit is the hormone-binding site and the β subunit contains the tyrosine kinase domain. *INSR* has 2 isoforms (exon 11 minus IR-A and exon 11 plus IR-B) by alternative splicing. Exon 11 encodes a carboxy-terminal sequence of 12 amino acids that is associated with more intense insulin binding.^[4,5]^ The binding of insulin to the α-subunit of IR triggers conformational changes that promote ATP binding, β-subunit phosphorylation, and the recruitment and subsequent phosphorylation of intracellular substrates, and also triggers the subsequent insulin signaling pathway.^[5]^

The extracellular portion of the monomer comprises 2 leucine-rich repeat domains (L1 and L2), a cysteine-rich region, and 3 fibronectin type III (FnIII) domains (FnIII-1–FnIII-3). Part of the insulin binding site of *INSR* and the α–β cleavage site are located within the FnIII domain.^[6]^ Hosoe et al showed that all missense mutations expected to severely impair hydrophobic core formation and FnIII structural domain stability cause DS, while all mutations expected to produce local instability and not affect FnIII structural domain folding cause RMS.^[7]^ Patients with severe *INSR* mutation-induced insulin resistance syndrome (DS or RMS) usually have a pure or compound heterozygous mutations in *INSR*. The patient in the present case showed compound heterozygous mutations for the novel variants c.2246delG and c.2646 + 5G>A. These 2 mutant loci had relatively little effect on insulin binding to the receptor, thus explaining the relatively mild phenotype, who is still alive at 11 years of age.

The patient presented with growth retardation, peculiar facial features, acanthosis nigricans, hypertrichosis, extremely high insulin levels, fasting hypoglycemia, and postprandial hyperglycemia. Acanthosis nigricans is a feature of severe insulin resistance. Several studies have suggested that this condition may be associated with high levels of circulating insulin cross-reacting with IGF-1 receptors on keratinocytes and dermal fibroblasts.^[8]^ Severe insulin resistance syndromes may manifest as hypertriglyceridemia, abnormal fat morphology, and adipose tissue loss.^[9]^ The patients in this study had normal triglyceride levels. Some patients may also have pineal abnormalities, and unfortunately, this patient did not have a pituitary MRI scan. An uncommon ocular complication (neovascular glaucoma) has also been reported in patients with RMS.^[10]^

As the disease progresses, insulin levels tend to decline in patients with RMS. These patients eventually develop persistent hyperglycemia, severe diabetic ketoacidosis, and diabetic microvascular complications including diabetic retinopathy and diabetic nephropathy.^[11,12]^ Therefore, in clinical practice, attention should be paid to the regular screening of patients by fundus photography, renal function, and urine protein-to-creatinine ratio to detect diabetic complications as early as possible and prevent disease progression.

Modern treatments for SIR are limited, and their outcomes are not promising. Proper diet and exercise are the basis of treatment. A low-fat diet is necessary in patients with severe hypertriglyceridemia. Metformin and thiazolidinediones play critical roles in improving insulin resistance and are often considered first-line treatment options and the first choice for multidrug therapy combinations.^[13]^ Insulin therapy should be initiated in cases in which the blood glucose levels are difficult to control. Eventually, very high doses of insulin are required, with multiple daily injections or continuous subcutaneous infusions of U-500 insulin often used as a viable treatment option.^[14,15]^ The IGF-1 receptor (IGF-1R) shares 45% to 65% structural homology with IR in the ligand-binding domain and 65% to 85% structural homology in the tyrosine kinase and substrate recruitment domains, resulting in a hybrid receptor IR/IGF-1R.^[5,16]^ Therefore, it may provide further treatment options for patients with severe insulin resistance syndrome. Early application of recombinant human IGF-1 (rhIGF-1) has been reported to prolong survival in patients with DS or RMS.^[17]^ Continuous subcutaneous rhIGF-1 (mecasermin) infusion via an insulin pump improved glucose homeostasis in a patient with DS and appeared to be more effective than a single injection.^[18]^ Because of its possible side effects and economic burden, mecasermin is still not approved and widely used; therefore, this drug should be used with full consideration of its therapeutic benefits. With the informed consent of the patient families, recombinant human methionyl leptin (metreleptin) can reduce BMI in SDS and improve glycemic control in patients with MRS.^[19,20]^ Evidence also suggests that sodium-glucose cotransporter 2 inhibitors (both dapagliflozin and empagliflozin) are well tolerated in patients with RMS, improving glycemic control without significantly increasing ketonemia; however, renal calcium excretion should be monitored regularly during the dosing phase.^[21,22]^ The treatment of SIR is challenging, and the therapeutic effect of these drugs appears to diminish over time; therefore, more clinical experience is needed to guide treatment.

The patient in the present case was characterized by fasting hypoglycemia and postprandial hyperglycemia; therefore, we advised him to consume sufficient carbohydrates at each meal, to perform appropriate physical activity after meals, and to ingest a small amount of protein such as eggs and milk before going to bed. We also advised him to take metformin orally at breakfast and lunch but not at dinner to reduce nocturnal hypoglycemia.

## 4. Conclusions

The phenotypes and genotypes of insulin resistance syndrome caused by mutations in *INSR* are heterogeneous. It is often difficult to distinguish between DS and RMS given their overlapping symptoms. We report the case of a male child with 2 novel *INSR* mutation loci (c.2246delG and c.2646 + 5G > A) identified by whole-exome genetic testing and diagnosed with SIR. In children who are not obese but with severe insulin resistance, growth retardation, hirsutism, and hyperglycemia, genetic testing should be performed for early diagnosis, active treatment, and follow-up. Other diseases that can lead to hyperinsulinemia must also be excluded, such as insulinoma and autoimmune insulin syndrome. However, modern treatments for SIR are limited; thus, more clinical experience is needed to guide clinical practice.

## Acknowledgments

The authors are very grateful to the patient for his kind contribution to this study. We also would like to thank Editage (www.editage.cn) for English language editing.

## Author contributions

**Conceptualization:** Lu Yu, Fang Yu, Shenglian Gan.

**Data curation:** Lu Yu.

**Formal analysis:** Lu Yu.

**Investigation:** Ting Sun, Qin Liu.

**Methodology:** Huilin Lu, Jian Luo.

**Project administration:** Shenglian Gan.

**Resources:** Fang Yu, Shenglian Gan.

**Validation:** Fang Yu, Shenglian Gan.

**Visualization:** Shenglian Gan.

**Writing—original draft:** Lu Yu.

**Writing—review and editing:** Zongrui Ma.
